# Association between mineral and bone disorder in patients with acute kidney injury following cardiac surgery and adverse outcomes

**DOI:** 10.1186/s12882-019-1572-y

**Published:** 2019-10-15

**Authors:** Tianye Yang, Wenji Wang, Xiao Tang, Peng Shi, Lulu Zhang, Wenyan Yu, Yingxin Xie, Daqiao Guo, Feng Ding

**Affiliations:** 1 Division of Nephrology & Critical Care Nephrology Unit, Shanghai Ninth People’s Hospital, School of Medicine, Shanghai Jiaotong University, 639 Zhizaoju Road, Shanghai, 200011 China; 2 Institute of Vascular surgery, Division of Vascular surgery, Zhongshan Hospital, Fudan University, Shanghai, 200032 China; 3 Department of Medical Statistics, Children’s Hospital, Center for Evidence-based Medicine, Fudan University, Shanghai, 200433 China

**Keywords:** Acute kidney injury, Cardiac surgery, Mineral and bone disorder, Adverse outcomes

## Abstract

**Background:**

Numerous studies have evaluated the prevalence and importance of mineral and bone disorders among patients with chronic kidney disease (CKD) and end-stage renal disease (ESRD). However, little is known about dysregulated mineral and bone metabolism in acute kidney injury (AKI).

**Methods:**

We evaluated the association between mineral and bone metabolites and clinical outcomes in 158 patients who underwent cardiac surgery and developed AKI between June 2014 and January 2016. The baseline characteristics of the patients were recorded, and the levels of mineral and bone metabolites, including calcium, phosphate, intact parathyroid hormone (iPTH), 25-hydroxyvitamin D (25D), bone-specific alkaline phosphatase (BAP), tartrate-resistant acid phosphatase 5b (TRACP-5b) and C-terminal fibroblast growth factor 23 (cFGF23) were measured within 12 h after establishing the clinical diagnosis.

**Results:**

The serum phosphate, iPTH and cFGF23 levels were significantly associated with the 28-day mortality (phosphate: Hazard Ratio [HR] =2.620, 95% CI: 1.083 to 6.338, *p* = 0.035; iPTH: HR = 1.044, 95% CI: 1.001 to 1.090, *p* = 0.046; cFGF23: HR = 1.367, 95% CI: 1.168 to 1.599, *p* < 0.001). Moreover, higher serum cFGF23 and BAP levels were independently associated with an increased risk of adverse outcomes. Additionally, we found that the serum cFGF23 levels rose most significantly and were associated with the severity of AKI (*P* < 0.001).

**Conclusions:**

Mineral and bone metabolites are dysregulated and are associated with adverse clinical outcomes among patients with AKI.

**Trial registration:**

www.clinicaltrials.gov NCT 00953992. Registered 6 August 2009.

## Background

Acute kidney injury (AKI) is a common problem in seriously ill patients and is associated with several poor clinical outcomes [1–6]. Numerous studies have proven that chronic kidney disease-mineral and bone disorder (CKD-MBD) is associated with adverse clinical outcomes [7–9]. In contrast, little is known about mineral and bone disorder and its association with outcomes among patients with AKI.

Reduced vitamin D levels, elevated parathyroid hormone (PTH) and fibroblast growth factor 23 (FGF23) levels and decreased or increased calcium and phosphate levels have been reported in patients with AKI in small studies [10–14], but their mechanisms are defined insufficiently. In the case of chronic kidney disease (CKD), the FGF23 level rises early in the course of the disease, stimulates urinary phosphate excretion and inhibits the activation of 25-hydroxyvitamin D (25D), and contributes to the development of secondary hyperparathyroidism [15].

In recent studies, the role of markers of bone metabolism in the prediction of mortality in patients with CKD has been prospectively evaluated. These studies have indicated a link between bone metabolism and cardiovascular events in patients with CKD. Markers of bone formation (bone-specific alkaline phosphatase [BAP]) and bone resorption (tartrate-resistant acid phosphatase 5b [TRACP-5b]) can serve as predictors of cardiovascular morbidity and mortality in patients with CKD [16].

Based on these observations, we hypothesized that the serum markers of mineral and bone metabolism can be used to predict the mortality of patients with AKI. To systematically evaluate the predictive value of the markers of mineral and bone metabolism for poor outcomes in patients with AKI, we designed a prospective cohort study to evaluate the predictive value of the markers of mineral and bone disorder for the mortality of patients who underwent cardiac surgery and developed AKI.

## Methods

### Study design

We conducted a single-center, prospective cohort study and enrolled patients who underwent cardiac surgery and developed incident AKI at a comprehensive hospital between June 2014 and January 2016. This study was approved by the hospital ethics committee of Shanghai Ninth People’s Hospital, School of Medicine, Shanghai Jiaotong University (approval number: [2014]45). All of the experimental protocols were implemented in accordance with the relevant guidelines and regulations. In accordance with the Declaration of Helsinki, patients or their legal guardians had obtained written informed consent prior to participation,. This study was registered at www.clinicaltrials.gov with the identification number NCT00953992.

### Study patients

The inclusion criteria were an age > 18 years and the development of incident AKI following cardiac surgery. AKI was defined as an increase in serum creatinine of ≥0.3 mg/dL within 48 h or that of ≥50% within 7 days, which conforme with the criteria established by the Kidney Disease Improving Global Outcomes (KDIGO) work group [17].

The exclusion criteria were: (1) preoperative AKI (defined as a 0.3 mg/dL rise in the serum creatinine level over 24 h or a 0.5 mg/dL rise over 48 h) [18], (2) pre-existing CKD (defined as an estimated glomerular filtration rate (eGFR) of < 30 mL/min per 1.73 m^2^) or ESRD requiring dialysis, (3) post-renal obstruction or rapid progressive glomerulonephritis as the main cause of AKI, (4) renal transplantation, (5) malignancy, and (6) pregnancy.

### Study procedures

The serum creatinine data of the participants were monitored daily in the hospital’s information system (HIS) to rapidly identify patients with incident AKI. We collected and stored the serum samples within 12 h after establishing the clinical diagnosis. The samples were centrifuged, aliquoted, and stored at − 80 °C within 2 h of collection.

### Clinical outcomes

We adjudicated all of the outcomes by reviewing the electronic medical records from the HIS. The pre-specified primary endpoint was the 28-day all-cause mortality.

The secondary endpoints were Renal Replacement Therapy or in-hospital mortality (RRT/death). Additional endpoints included the ventilator-free days and hospital-free days in 28 days. The patients who died before 28 days were graded a score of zero.

### Laboratory assessment

The bone-specific alkaline phosphatase and tartrate-resistant acid phosphatase 5b levels were measured using a standard ELISA kit (Immunodiagnostic Systems Limited, UK). The C-terminal FGF-23 levels were measured using a standard ELISA kit (Immutopics Inc., USA). The serum calcium and phosphate levels were measured on an automated chemistry analyzer (AU5800, Beckman Coulter, USA). The iPTH level was measured on an automated immunoassay analyzer (UniCel DxI800, Beckman Coulter, USA). The 25-hydroxyvitamin D level was measured on an automated immunoassay analyzer (LIAISON, DiaSorin, Italy).

### Statistical analyses

The data are reported as the median and interquartile range (IQR: 25th percentile–75th percentile) for continuous variables and as the *n* (%) for categorical variables. The baseline and operative characteristics were compared in patients with different stages of AKI using the Kruskal-Wallis test for continuous variables with a non-normal distribution and using the chi-square test for category variables. The correlation between the enrollment mineral metabolite levels and the other variables was analyzed using the Spearman rank correlation coefficient. The comparison of the mineral metabolite levels among the patients who died and those of the patients who did not die was assessed using the Mann-Whitney *U* test.

Univariate Cox regression was used to identify potential confounding variables, including demographics (sex and age), preoperative renal function (serum creatintine and eGFR), comorbidities (hypertension, chronic heart failure and diabetes mellitus), operative characteristics (operation type, whether cardiopulmonary bypass or not), postoperative characteristics (low blood pressure, applying diuretics), Acute Physiology and Chronic Health Evaluation (APACHE) II score and biochemical parameters. After that, we used multivariate Cox regression to adjust for covariates using two different models: model 1 was adjusted for age, sex, baseline eGFR, hypertension, chronic heart failure (New York Heart Association functional class [NYHA] ≥ 2) and diabetes mellitus; model 2 was further adjusted for the operation type and APACHE II score on enrollment, according to *P* value in the univariable regression (*P* < 0.1) or clinical importance. Logistic and linear regression models were used to assess the association between the mineral metabolite levels and secondary endpoints, adjusting for the same covariates as above. Receiver-operating characteristics (ROC) curves and areas under the curves (AUCs) were calculated using the survival and time ROC packages to compare the predictability of the mineral and bone metabolite levels for the 28-day mortality, Furthermore, the C-index was calculated using the survival package. The evaluation criterion of the C-index was similar to that of the AUC.

We used linear mixed models for the repeated measures to test for significant differences in the mineral and bone metabolite levels over time between the patients with different severities of AKI. The comparison of the mineral and bone metabolite levels at individual time points was assessed using the Spearman rank correlation coefficient (for the correlation between the severity of AKI and the mineral and bone metabolite levels).

All of the comparisons were two-tailed, with *P* < 0.05 considered as being statistically significant. The statistical analysis was performed using the software package R version 3.4.0 (www.r-project.org).

## Results

### Patient characteristics

We enrolled 158 patients who developed incident AKI following cardiac surgery but had no evidence of AKI at baseline into a prospective cohort study.

Among the 158 patients enrolled, 57 (36.1%) had AKI stage 1, 53 (33.5%) had AKI stage 2, and 48 (30.4%) had AKI stage 3. The median (interquartile range [IQR]) age of the patients was 57 years (49–66 years), and 74.7% of them were men. The most common comorbidities were congestive heart failure [NYHA≥2] (62.0%), hypertension (53.8%), and diabetes mellitus (10.1%). The median (IQR) APACHE II score was 11 (9–13). The additional baseline characteristics are shown in Table 1.

**Table 1:** Baseline and operative characteristics according to the 3 different stages of AKI in 158 patients

	AKI (*n* = 158)	Stage 1 (*n* = 57)	Stage 2 (*n* = 53)	Stage 3 (*n* = 48)	*P* value
Demographics					
Male	118 (74.7)	43 (75.4)	40 (75.5)	35 (72.9)	0.945
Age (yrs)	57 (49–66)	58 (50–67)	58 (50–66)	56 (41–65)	0.223
Preoperative renal function					
Serum creatinine (μmol/L)	81 (71–90)	85 (75–94)	80 (70–88)	78 (67–90)	0.220
eGFR (mL/min/1.73 m^2^)	87 (73–100)	83 (72–94)	88 (74–100)	91 (72–106)	0.083
≥ 30 and < 60	6 (3.8)	4 (7.0)	1 (1.9)	1 (2.1)	0.294
≥ 60 and < 90	84 (53.2)	33 (57.9)	29 (54.9)	22 (45.8)	
≥ 90	68 (43.0)	20 (35.1)	23 (43.4)	25 (52.1)	
Comorbidities					
Hypertension	85 (53.8)	25 (43.9)	34 (64.2)	26 (54.2)	0.103
NYHA≥2	98 (62.0)	34 (59.6)	35 (66.0)	29 (60.4)	0.759
Diabetes mellitus	16 (10.1)	6 (10.5)	5 (9.4)	5 (10.4)	0.979
Type of procedure					
Valve alone	74 (46.8)	30 (52.6)	23 (43.4)	21 (43.8)	0.548
CABG alone	23 (14.6)	13 (22.8)	9 (17.0)	1 (2.1)	0.009
CABG and valve	13 (8.2)	7 (12.3)	4 (7.5)	2 (4.2)	0.313
Aortic dissection	35 (22.2)	5 (8.8)	12 (22.6)	18 (37.5)	0.002
CPB	93 (58.9)	33 (57.9)	30 (56.6)	30 (62.5)	0.820
Postoperative characteristics					
Low blood pressure	90 (57.0)	21 (36.8)	26 (49.1)	43 (89.6)	< 0.001
Applying diuretics	131 (82.9)	48 (84.2)	47 (88.7)	36 (75.0)	0.180
Severity of illness					
APACHE II score	11 (9–13)	10 (8–12)	11 (9–13)	12 (9–15)	0.007

The data are presented as the *n* (%) or median (interquartile range [IQR]: 25th–75th percentile). The P value was computed for global comparisons among the groups using the Kruskal-Wallis and chi-square tests for the continuous and categorical variables, respectively. AKI: acute kidney injury; eGFR: estimated glomerular filtration rate; NYHA: New York Heart Association class; CABG: coronary artery bypass grafting; CPB: cardiopulmonary bypass; APACHE: Acute Physiology and Chronic Health Evaluation. The eGFR was determined using the Modification of Diet in Renal Disease equation. The APACHE II score is an ICU severity of illness scoring system ranging from 0 to 71, with higher scores indicating a more severe disease

Among these three groups, the patients with aortic dissection and those with low blood pressure after the operation presented with higher ratios of severe kidney injury (*P* = 0.002 and *P* < 0.001, respectively). Meanwhile, the ratios of AKI stage 3 were lowest in the patients with coronary artery bypass alone (*P* = 0.009). Moreover, patients with more severe kidney injury had higher scores of the APACHE II (*P* = 0.007). However, there were no significant differences among three groups in the demographics, preoperative renal function, comorbidities, cardiopulmonary bypass during the operation, and applying diuretics after the operation (*P* > 0.05).

### Serum mineral metabolite correlations

The serum iPTH level was inversely correlated with the corrected calcium level (rs = − 0.379, *P* < 0.001) and directly correlated with the cFGF23 level (rs =0.330, P < 0.001). The serum TRACP-5b level was directly correlated with the 25D level (rs =0.189, *P* < 0.05) and BAP level (rs =0.179, P < 0.05). The additional correlations are shown in Additional file 1: Table S1.

### Serum mineral metabolite levels and their relationship with in-hospital mortality at 28 days

Among the 158 patients enrolled in this study, 18 (18.8%) had died within 28 days, and the median time from the clinical diagnosis to death was 7 days (IQR: 2–17 days). The causes of death included multiple organ dysfunction syndromes (*n* = 7), heart failure (*n* = 4), adult respiratory distress syndrome (*n* = 3), acute cerebral infarction (*n* = 2), cute gangrenous cholecystitis (*n* = 1) and circulatory failure of unknown disease (n = 1). The serum mineral metabolite levels in patients who died and in those who did not die are shown in Table 2. The patients who died had higher levels of serum cFGF23 (*P* = 0.007), as well as lower levels of 25D (slightly insignificant, *P* = 0.072), compared with the patients who were alive. The levels of the other mineral metabolites were similar between the two groups (Table 2).

**Table 2:** Serum mineral metabolite levels in the patients who died and in those who did not die

	Alive (*n* = 140)	Dead (*n* = 18)	*P* value
Calcium (mmol/L)	2.07 (1.94–2.19)	2.05 (1.96–2.23)	0.840
Phosphate (mmol/L)	1.08 (0.75–1.41)	1.03 (0.86–1.55)	0.665
iPTH (ng/dL)	11.8 (7.3–16.5)	15.2 (10.2–18.5)	0.185
25D (ng/mL)	16.7 (12.4–21.7)	14.5 (12.2–16.2)	0.072
BAP (μg/L)	8.48 (5.88–12.69)	7.95 (7.06–16.81)	0.494
TRACP-5b (U/L)	2.06 (1.34–2.84)	2.50 (1.30–3.53)	0.298
cFGF23 (RU/μL)	0.223 (0.069–0.534)	0.813 (0.188–4.488)	0.007

The data are presented as the median (interquartile range [IQR]: 25th–75th percentile). iPTH: intact parathyroid hormone; 25D: 25-hydroxyvitamin D; BAP: bone-specific alkaline phosphatase; TRACP-5b: tartrate-resistant acid phosphatase 5b; cFGF23: C-terminal fibroblast growth factor 23

Then, we analyzed the association between each mineral metabolite biomarker and the risk of death after AKI. Table 3 shows the unadjusted and adjusted hazard ratios for the 28-day mortality risk according to the levels of the mineral metabolites. In the unadjusted analyses, the serum iPTH and cFGF23 levels were both significantly associated with the 28-day mortality (iPTH: HR = 1.045, 95% CI: 1.010 to 1.080, *p* = 0.009; cFGF23: HR = 1.258, 95% CI: 1.149 to 1.378, *p* < 0.001). These associations were slightly attenuated but remained significant when the model was adjusted for age, sex, preoperative eGFR, hypertension, congestive heart failure, and diabetes mellitus (Model 1), as well as when it was further adjusted for operation type and APACHE II score (Model 2; iPTH: HR = 1.044, 95% CI: 1.001 to 1.090, *p* = 0.046; cFGF23: HR = 1.367, 95% CI: 1.168 to 1.599, p < 0.001). The serum phosphate level was also associated with the 28-day mortality in the adjusted analyses (HR = 2.620, 95% CI: 1.083 to 6.338, *p* = 0.035). The other biomarkers, including the corrected calcium, 25D, BAP, and TRACP-5b, were not associated with the 28-day mortality. Additionally, Table 4 shows the area under the receiver operating characteristic for the 28-day mortality risk according to the levels of the mineral metabolites. The strengths of the AUC appeared to be greater for cFGF23 than for the other mineral biomarkers that were tested (*P* = 0.007).

**Table 3:** Hazard ratios (HRs) and 95% confidence intervals (CIs) of the 28-day mortality according to the mineral metabolite levels in all 158 patients with AKI

Biomarker	Unadjusted		Model 1		Model 2	
	Hazard ratio (95% CI)	P value	Hazard ratio (95% CI)	P value	Hazard ratio (95% CI)	*P* value
Calcium (mmol/L)	1.42 (0.21, 9.63)	0.710	2.08 (0.30, 14.36)	0.464	2.29 (0.29, 17.90)	0.434
Phosphate (mmol/L)	1.73 (0.72, 4.17)	0.227	2.01 (0.87, 4.68)	0.108	2.62 (1.08, 6.34)	0.035
iPTH (ng/dL)	1.05 (1.01, 1.08)	0.009	1.05 (1.01, 1.08)	0.018	1.04 (1.00, 1.09)	0.046
25D (ng/mL)	0.93 (0.86, 1.01)	0.080	0.93 (0.86, 1.01)	0.091	0.94 (0.86, 1.02)	0.157
BAP (μg/L)	1.03 (0.96, 1.10)	0.395	1.03 (0.96, 1.11)	0.362	1.05 (0.97, 1.13)	0.190
TRACP-5b (U/L)	1.26 (0.83, 1.91)	0.283	1.18 (0.75, 1.83)	0.484	1.37 (0.84, 2.22)	0.206
cFGF23 (RU/μL)	1.26 (1.15, 1.38)	< 0.001	1.37 (1.19, 1.58)	< 0.001	1.37 (1.17, 1.60)	< 0.001

Model 1 is adjusted for age, sex, preoperative eGFR, hypertension, congestive heart failure, and diabetes mellitus. Model 2 is further adjusted for the operation type and APACHE II score. iPTH: intact parathyroid hormone; 25D: 25-hydroxyvitamin D; BAP: bone-specific alkaline phosphatase; TRACP-5b: tartrate-resistant acid phosphatase 5b; cFGF23: C-terminal fibroblast growth factor 23; eGFR: estimated glomerular filtration rate; APACHE: Acute Physiology and Chronic Health Evaluation

**Table 4:** Receiver operating characteristic for the 28-day mortality risk according to the levels of the mineral metabolites

Biomarker	AUC ROC	C index	*P* Value
Calcium	0.422	0.540	0.584
Phosphate	0.546	0.532	0.662
iPTH	0.652	0.597	0.149
25D	0.371	0.619	0.029
BAP	0.610	0.557	0.386
TRACP-5b	0.574	0.571	0.368
cFGF23	0.688	0.697	0.007

*AUC ROC* area under the receiver operating characteristic curve, *C index* index of concordance, *iPTH* intact parathyroid hormone, *25D* 25-hydroxyvitamin D, *BAP* bone-specific alkaline phosphatase, *TRACP-5b* tartrate-resistant acid phosphatase 5b, *cFGF23* C-terminal fibroblast growth factor 23

In an exploratory analysis, we evaluated whether the phosphate, iPTH, and cFGF23 levels remained associated with 28-day mortality after the adjustment for each. As shown in Additional file 1: Table S2, only the cFGF23 level, and not the iPTH or phosphate levels, remained as being significantly associated with the 28-day mortality even after the adjustment for each (HR = 1.380, 95% CI: 1.162 to 1.639, *p* < 0.001).

### Serum mineral metabolite levels and other adverse outcomes

Table 5 shows the adjusted logistic and linear regression models of the secondary endpoints according to the levels of the mineral metabolites. The serum cFGF23 levels were also associated with the following secondary endpoint in the adjusted analyses: RRT/death (OR = 1.469, 95% CI: 1.055 to 2.045, *p* = 0.029). Additionally, the serum cFGF23 and BAP levels were inversely associated with the hospital-free days and ventilator-free days, implying that they were correlated with longer durations of hospitalization and mechanical ventilation (hospital-free days: cFGF23, β [SEM]: − 0.951 [0.264], *p* < 0.001; BAP, β [SEM]: − 0.299 [0.087], p < 0.001; ventilator-free days: cFGF23, β [SEM]: − 1.681 [0.365], p < 0.001; BAP, β [SEM]: − 0.268 [0.126], *p* = 0.027).

**Table 5:** Multivariable logistic and linear regression models of the secondary endpoints according to the mineral metabolite levels in all 158 patients with AKI

Biomarker	RRT/Death		Hospital-free days		Ventilator-free days	
	OR (95% CI)	P value	β (SEM)	P value	β (SEM)	*P* value
Calcium (mmol/L)	5.57 (0.66, 47.32)	0.111	−3.19 (2.54)	0.203	−1.45 (3.62)	0.721
Phosphate (mmol/L)	0.94 (0.42, 2.10)	0.973	−0.66 (1.09)	0.580	−0.46 (1.55)	0.852
iPTH (ng/dL)	1.00 (0.97, 1.04)	0.935	−0.05 (0.06)	0.388	−0.16 (0.08)	0.031
25D (ng/mL)	0.97 (0.90, 1.04)	0.355	−0.16 (0.08)	0.050	−0.13 (0.12)	0.288
BAP (μg/L)	1.07 (1.00, 1.16)	0.073	−0.30 (0.09)	< 0.001	−0.27 (0.13)	0.027
TRACP-5b (U/L)	1.21 (0.77, 1.92)	0.413	−0.13 (0.54)	0.819	−1.07 (0.76)	0.148
cFGF23 (RU/μL)	1.47 (1.06, 2.05)	0.029	−0.95 (0.26)	< 0.001	−1.68 (0.37)	< 0.001

*AKI* acute kidney injury, *RRT/death* renal replacement therapy or in-hospital mortality, *iPTH* intact parathyroid hormone, *25D* 25-hydroxyvitamin D, *BAP* bone-specific alkaline phosphatase, *TRACP-5b* tartrate-resistant acid phosphatase 5b, *cFGF23* C-terminal fibroblast growth factor 23

### Kinetics of the serum mineral metabolite levels in AKI after cardiac surgery

**Fig. 1:**
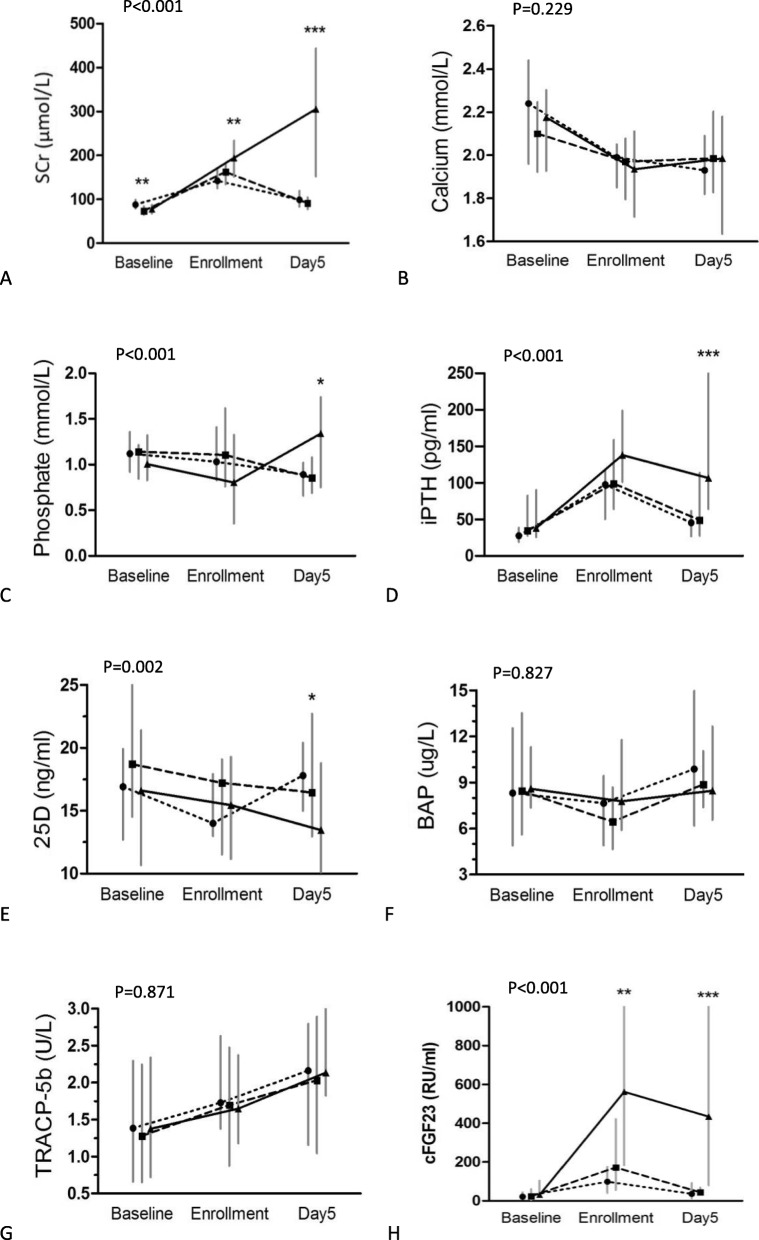
Mineral and bone metabolite levels in a subgroup of 71 patients with different stages of AKI. The (**a**) Scr levels, (**b**) calcium levels, (**c**) phosphate levels, (**d**) iPTH levels, (**e**) 25D levels, (**f**) BAP levels, (**g**) TRACP-5b levels, and (**h**) cFGF23 levels. --●-- (dot): AKI stage 1 (*n* = 23); −-▇--(square): AKI stage 2 (*n* = 24); ▲ (triangle): AKI stage 3 (*n* = 24). *P* values are from repeated-measures linear mixed models comparing the 3 groups. *P < 0.05, **P < 0.01, and ***P < 0.001; a comparison of the biomarker levels cross AKI categories at individual time points. The data are shown as the median (interquartile range). Scr: serum creatinine; iPTH: intact parathyroid hormone; 25D: 25-hydroxyvitamin D; BAP: bone-specific alkaline phosphatase; TRACP-5b: tartrate-resistant acid phosphatase 5b; cFGF23: C-terminal fibroblast growth factor 23

To compare the time course of changes in the mineral and bone metabolite biomarkers in patients with AKI after cardiosurgery, we also measured the levels in a subgroup of 71 patients, and these results are shown in Fig. 1. We found that there was a hierarchical association between the AKI severity and the levels of cFGF23 over time (*P* < 0.001). Among the AKI stage 3 patients, we found rapid and significant increases in the cFGF23 levels like that the median levels at enrollment were ~ 18-fold higher than the baseline (preoperative) levels (P < 0.001) and the Day 5 levels were ~ 14-fold higher (P < 0.001) than the baseline levels. Among the AKI stage 1 patients, the median cFGF23 levels increased by ~ 4.5-fold at the time of enrollment and by ~ 1.6-fold on Day 5. The AKI stage 2 group of patientshad medium rises in the cFGF23 levels that were between those of the AKI stage 1 group and those of the AKI stage 3 group. The serum creatinine and iPTH levels showed a similar hierarchical association, which higher levels were associated with a greater severity of AKI. Additionally, when the phosphate and 25D levels were evaluated at individual time points, only the Day 5 levels were significantly different between the groups. The longitudinal calcium levels, as well as the BAP and TRACP-5b levels, were similar in these three groups.

## Discussion

In this prospective cohort study, we confirmed that higher serum cFGF23 levels obtained within 12 h of a clinical diagnosis for AKI after cardiac surgery are independently associated with a greater 28-day mortality risk, as well as with several other adverse outcomes, including in-hospital mortality and the need for RRT, and fewer ventilator-free and hospital-free days. Additionally, we also observed that other mineral and bone metabolites, such as phosphate, iPTH, and BAP, are marginally associated with poor outcomes. Moreover, we found that the serum cFGF23 levels rose most significantly and were associated with the severity of AKI. These findings suggest that there is a complex interplay between AKI, mineral and bone metabolism, and adverse clinical outcomes after cardiac surgery.

Multiple studies have demonstrated that mineral and bone disorders are prevalent and associated with mortality in patients with CKD and ESRD [7–9]. However, studies on minerals, particularly those on bone metabolite levels in AKI, are limited. In contrast to our prior study, which investigated the levels of serum vitamin D (including 1,25-dihydroxyvitamin D and 25-hydroxyvitamin D) in patients with hospital-acquired AKI only [14], here, we evaluated both mineral and bone metabolite levels in patients with AKI after cardiac surgery. Thus, the findings from this study not only demonstrate an independent and prospective association between the mineral metabolite levels and poor outcomes of AKI but also represent the comprehensive study of bone metabolites in clinical setting, which is a rare finding in similar AKI cohort studies.

BAP and TRACP-5b are sensitive biomarkers of osteoblastic bone formation and osteoclastic bone resorption [8, 19, 20]. The few prior studies that measured the serum BAP and TRACP-5b levels were conducted almost exclusively among patients with CKD or ESRD. Astrid et al. measured the levels of bone markers in serum samples from 627 patients with CKD and found that the markers of bone formation (BAP: OR: 1.01, 95% CI: 1.01–1.02) and bone resorption (TRACP-5b: OR: 0.86, 95% CI: 0.75–0.99) can serve as predictors of cardiovascular morbidity and mortality in patients with CKD [16]. Further contrasting with the results of prior studies, our data indicated that a high BAP serum level, which implies that there is high bone formative activity, is potentially associated with an increased risk of death in patients with AKI.

In addition to the 28-day mortality, we also evaluated the association between the mineral and bone metabolite levels and several secondary endpoints. The serum cFGF23 levels were markedly and independently associated with RRT/Death and longer durations of mechanical ventilation and hospital length of stay. Coincidentally, higher serum BAP levels were also associated with longer durations of mechanical ventilation and hospital length of stay.

Whether mineral and bone metabolite levels are merely an illness severity marker or directly contribute to poor outcomes is a critical question that could not be answered by this study. Higher FGF23 levels are related to pathogenesis of left ventricular hypertrophy [21–23] and inflammation [24–26] in CKD. In fact, a large number of epidemiologic data suggest that decreased vitamin D metabolite levels are associated with adverse outcomes in those with critical illness [27, 28]. Maybe increased FGF23 levels could lead to adverse outcomes via inhibiting the activation of vitamin D metabolite [15, 26]. However, we did not find positive or inverse correlations between the serum cFGF23 and 25D levels, and we also did not find a correlation between the 25D level and the 28-mortality risk, which is consistent with the results of our previous study. Thus, the associations between the FGF23 and vitamin D metabolite levels remain unclear.

We acknowledge several limitations of this study. All samples used in our study were from one single centre. Therefore, selection bias in the enrollment was inevitable. 18 patients had died within 28 days, due to the limitation of the sample size in this cohort study, no further subgroup analysis has been done for the cause of death, and so it was difficult to determine the link between mineral and bone disorder and the cause of death. Additionally, urine output was an important criterion, which was not accessed in this study. Thus, the incidence of AKI may be underestimated and the estimation of AKI stages may be affected. Of course, this result was only uncovered in a small sample size and a larger clinical sample is needed to confirm this result.

## Conclusions

The serum cFGF23 levels measured within 12 h of the AKI clinical diagnosis are independently associated with the 28-day mortality and other adverse outcomes of patients with AKI after undergoing cardiac surgery. Other mineral and bone metabolites, including phosphate, iPTH, and BAP, are marginally associated with poor outcomes.

## Supplementary information

**Additional file 1: Table S1.** Spearman correlations for all of the mineral metabolites in 158 patients with AKI. **Table S2.** Hazard ratios (HRs) and 95% confidence intervals (CIs) of the 28-day mortality according to the phosphate, iPTH and cFGF23 levels (after the adjustment for each one).

## Data Availability

The datasets analysed during the present study are available from the corresponding author on reasonable request.

## Abbreviations

25D: 25-hydroxyvitamin D
AKI: Acute kidney injury
APACHE: Acute Physiology and Chronic Health Evaluation
BAP: Bone-specific alkaline phosphatase
cFGF23: C-terminal fibroblast growth factor 23
CKD: Chronic kidney disease
CKD-MBD: Chronic kidney disease-mineral and bone disorder
eGFR: Estimated glomerular filtration rate
ESRD: End-stage renal disease
HIS: Hospital’s information system
iPTH: Intact parathyroid hormone
KDIGO: Kidney Disease Improving Global Outcomes
NYHA: New York Heart Association
RRT: Renal Replacement Therapy
TRACP-5b: Tartrate-resistant acid phosphatase 5b

## References

[1] Schrier RW, Wang W, Poole B, Mitra A (2004). Acute renal failure: definitions, diagnosis, pathogenesis, and therapy. J Clin Invest.

[2] Loef BG, Epema AH, Smilde TD, Henning RH, Ebels T, Navis G, Stegeman CA (2005). Immediate postoperative renal function deterioration in cardiac surgical patients predicts in-hospital mortality and long-term survival. J Am Soc Nephrol.

[3] Chertow GM, Burdick E, Honour M, Bonventre JV, Bates DW (2005). Acute kidney injury, mortality, length of stay, and costs in hospitalized patients. J Am Soc Nephrol.

[4] Yang L, Xing G, Wang L, Wu Y, Li S, Xu G, He Q, Chen J, Chen M, Liu X, Zhu Z, Yang L, Lian X, Ding F, Li Y, Wang H, Wang J, Wang R, Mei C, Xu J, Li R, Cao J, Zhang L, Wang Y, Xu J, Bao B, Liu B, Chen H, Li S, Zha Y, Luo Q, Chen D, Shen Y, Liao Y, Zhang Z, Wang X, Zhang K, Liu L, Mao P, Guo C, Li J, Wang Z, Bai S, Shi S, Wang Y, Wang J, Liu Z, Wang F, Huang D, Wang S, Ge S, Shen Q, Zhang P, Wu L, Pan M, Zou X, Zhu P, Zhao J, Zhou M, Yang L, Hu W, Wang J, Liu B, Zhang T, Han J, Wen T, Zhao M, Wang H, Consortiums IAbC (2015). Acute kidney injury in china: A cross-sectional survey. Lancet.

[5] Druml W, Lenz K, Laggner AN (2015). Our paper 20 years later: from acute renal failure to acute kidney injury--the metamorphosis of a syndrome. Intens Care Med.

[6] Uchino S, Kellum JA, Bellomo R, Doig GS, Morimatsu H, Morgera S, Schetz M, Tan I, Bouman C, Macedo E, Gibney N, Tolwani A, Ronco C (2005). Beginning, ending supportive therapy for the kidney I: acute renal failure in critically ill patients: a multinational, multicenter study. Jama.

[7] Kestenbaum B, Sampson JN, Rudser KD, Patterson DJ, Seliger SL, Young B, Sherrard DJ, Andress DL (2005). Serum phosphate levels and mortality risk among people with chronic kidney disease. J Am Soc Nephrol.

[8] Kidney Disease: Improving Global Outcomes CKD-MBD Work Group: Kdigo clinical practice guideline for the diagnosis, evaluation, prevention, and treatment of chronic kidney disease-mineral and bone disorder (ckd-mbd). Kidney Int Suppl. 2009:S1-130. 10.1038/ki.2009.188 19644521

[9] Palmer SC, Hayen A, Macaskill P, Pellegrini F, Craig JC, Elder GJ, Strippoli GF (2011). Serum levels of phosphorus, parathyroid hormone, and calcium and risks of death and cardiovascular disease in individuals with chronic kidney disease: a systematic review and meta-analysis. Jama.

[10] Llach F, Felsenfeld AJ, Haussler MR (1981). The pathophysiology of altered calcium metabolism in rhabdomyolysis-induced acute renal failure. Interactions of parathyroid hormone, 25-hydroxycholecalciferol, and 1,25-dihydroxycholecalciferol. New Engl J Med.

[11] Leaf DE, Waikar SS, Wolf M, Cremers S, Bhan I, Stern L (2013). Dysregulated mineral metabolism in patients with acute kidney injury and risk of adverse outcomes. Clin Endocrinol.

[12] Leaf DE, Jacob KA, Srivastava A, Chen ME, Christov M, Juppner H, Sabbisetti VS, Martin A, Wolf M, Waikar SS: Fibroblast growth factor 23 levels associate with aki and death in critical illness. J Am Soc Nephrol. 2017;28(6):1877-85. 10.1681/ASN.2016080836 PMC5461795 28028134

[13] Leaf DE, Christov M, Juppner H, Siew E, Ikizler TA, Bian A, Chen G, Sabbisetti VS, Bonventre JV, Cai X, Wolf M, Waikar SS (2016). Fibroblast growth factor 23 levels are elevated and associated with severe acute kidney injury and death following cardiac surgery. Kidney Int.

[14] Lai L, Qian J, Yang Y, Xie Q, You H, Zhou Y, Ma S, Hao C, Gu Y, Ding F (2013). Is the serum vitamin d level at the time of hospital-acquired acute kidney injury diagnosis associated with prognosis?. PLoS One.

[15] van Husen M, Fischer AK, Lehnhardt A, Klaassen I, Moller K, Muller-Wiefel DE, Kemper MJ (2010). Fibroblast growth factor 23 and bone metabolism in children with chronic kidney disease. Kidney Int.

[16] Fahrleitner-Pammer A, Herberth J, Browning SR, Obermayer-Pietsch B, Wirnsberger G, Holzer H, Dobnig H, Malluche HH (2008). Bone markers predict cardiovascular events in chronic kidney disease. J Bone Miner Res.

[17] Khwaja A (2012). Kdigo clinical practice guidelines for acute kidney injury. Nephron Clin Pract.

[18] Waikar SS, Bonventre JV (2009). Creatinine kinetics and the definition of acute kidney injury. J Am Soc Nephrol.

[19] Yamada S, Inaba M, Kurajoh M, Shidara K, Imanishi Y, Ishimura E, Nishizawa Y (2008). Utility of serum tartrate-resistant acid phosphatase (tracp5b) as a bone resorption marker in patients with chronic kidney disease: Independence from renal dysfunction. Clin Endocrinol.

[20] Halleen JM, Tiitinen SL, Ylipahkala H, Fagerlund KM, Vaananen HK (2006). Tartrate-resistant acid phosphatase 5b (tracp 5b) as a marker of bone resorption. Clin Lab.

[21] Grabner A, Amaral AP, Schramm K, Singh S, Sloan A, Yanucil C, Li J, Shehadeh LA, Hare JM, David V, Martin A, Fornoni A, Di Marco GS, Kentrup D, Reuter S, Mayer AB, Pavenstadt H, Stypmann J, Kuhn C, Hille S, Frey N, Leifheit-Nestler M, Richter B, Haffner D, Abraham R, Bange J, Sperl B, Ullrich A, Brand M, Wolf M, Faul C (2015). Activation of cardiac fibroblast growth factor receptor 4 causes left ventricular hypertrophy. Cell Metab.

[22] Mathew JS, Sachs MC, Katz R, Patton KK, Heckbert SR, Hoofnagle AN, Alonso A, Chonchol M, Deo R, Ix JH, Siscovick DS, Kestenbaum B, de Boer IH (2014). Fibroblast growth factor-23 and incident atrial fibrillation: the multi-ethnic study of atherosclerosis (mesa) and the cardiovascular health study (chs). Circulation.

[23] Faul C, Amaral AP, Oskouei B, Hu MC, Sloan A, Isakova T, Gutierrez OM, Aguillon-Prada R, Lincoln J, Hare JM, Mundel P, Morales A, Scialla J, Fischer M, Soliman EZ, Chen J, Go AS, Rosas SE, Nessel L, Townsend RR, Feldman HI, St John Sutton M, Ojo A, Gadegbeku C, Di Marco GS, Reuter S, Kentrup D, Tiemann K, Brand M, Hill JA, Moe OW, Kuro OM, Kusek JW, Keane MG, Wolf M (2011). Fgf23 induces left ventricular hypertrophy. J Clin Invest.

[24] Munoz Mendoza J, Isakova T, Cai X, Bayes LY, Faul C, Scialla JJ, Lash JP, Chen J, He J, Navaneethan S, Negrea L, Rosas SE, Kretzler M, Nessel L, Xie D, Anderson AH, Raj DS, Wolf M, Investigators CS (2017). Inflammation and elevated levels of fibroblast growth factor 23 are independent risk factors for death in chronic kidney disease. Kidney Int.

[25] Hanudel M, Juppner H, Salusky IB (2016). Fibroblast growth factor 23: fueling the fire. Kidney Int.

[26] Chonchol M, Greene T, Zhang Y, Hoofnagle AN, Cheung AK (2016). Low vitamin d and high fibroblast growth factor 23 serum levels associate with infectious and cardiac deaths in the hemo study. J Am Soc Nephrol.

[27] Annweiler C, Pochic S, Fantino B, Legrand E, Bataille R, Montero-Odasso M, Beauchet O (2010). Serum vitamin d concentration and short-term mortality among geriatric inpatients in acute care settings. Adv Ther.

[28] Saha H, Mustonen J, Pietila K, Pasternack A (1993). Metabolism of calcium and vitamin d3 in patients with acute tubulointerstitial nephritis: a study of 41 patients with nephropathia epidemica. Nephron.

