# Supplementary material for: Association between mineral and bone disorder in patients with acute kidney injury following cardiac surgery and adverse outcomes
Source: BMC Nephrol. 2019 Oct 15;20:369. doi: 10.1186/s12882-019-1572-y (PMC6794865; doi:10.1186/s12882-019-1572-y)
Supplement: Supplementary file 1 — Additional file 1: Table S1. Spearman correlations for all of the mineral metabolites in 158 patients with AKI. Table S2. Hazard ratios (HRs) and 95% confidence intervals (CIs) of the 28-day mortality according to the phosphate, iPTH and cFGF23 levels (after the adjustment for each one). [file 12882_2019_1572_MOESM1_ESM.docx]

**Additional file 1:Table S1. Spearman correlations for all of the mineral metabolites in 158 patients with AKI.**

|  | Phosphate | iPTH | 25D | BAP | TRACP-5b | cFGF23 |
| --- | --- | --- | --- | --- | --- | --- |
| Calcium | 0.012 | -0.379*** | -0.042 | -0.115 | -0.104 | 0.031 |
| Phosphate | － | -0.040 | -0.028 | 0.002 | 0.131 | -0.100 |
| iPTH | － | － | -0.024 | -0.031 | 0.147 | 0.330*** |
| 25D | － | － | － | 0.106 | 0.189* | -0.153 |
| BAP | － | － | － | － | 0.179* | 0.026 |
| TRACP-5b | － | － | － | － | － | -0.036 |

iPTH: intact parathyroid hormone; 25D: 25-hydroxyvitamin D; BAP: bone-specific alkaline phosphatase; TRACP-5b: tartrate-resistant acid phosphatase 5b; cFGF23: C-terminal fibroblast growth factor 23; *: P<0.05; **: P<0.01; ***: P<0.001.

**Table S2. Hazard ratios (HRs) and 95% confidence intervals (CIs) of the 28-day mortality according to the phosphate, iPTH and cFGF23 levels (after the adjustment for each one).**

|  | Model 1 | Model 2 |
| --- | --- | --- |
| Biomarker | Hazard ratio (95% CI) P value | Hazard ratio (95% CI) P value |
| Phosphate (mmol/L) | 2.62 (1.08, 6.34) 0.035 | 2.52 (1.00, 6.34) 0.066 |
| iPTH (ng/dL) | 1.04 (1.00, 1.09) 0.046 | 1.01 (0.97, 1.06) 0.616 |
| cFGF23 (RU/µL) | 1.37 (1.17, 1.60) <0.001 | 1.38 (1.16, 1.64) <0.001 |

Model 1 is adjusted for age, sex, preoperative eGFR, hypertension, congestive heart failure, diabetes mellitus, operation type, and APACHE II score. Model 2 is further adjusted for phosphate, iPTH and cFGF23. iPTH: intact parathyroid hormone; cFGF23: C-terminal fibroblast growth factor 23; eGFR: estimated glomerular filtration rate; APACHE: Acute Physiology and Chronic Health Evaluation.
